# The value of urinary exosomal lncRNA SNHG16 as a diagnostic biomarker for bladder cancer

**DOI:** 10.1007/s11033-023-08667-z

**Published:** 2023-08-17

**Authors:** Chengyi Liu, Pengcheng Xu, Song Shao, Fang Wang, Zhiwen Zheng, Shuangjie Li, Wei Liu, Guangyuan Li

**Affiliations:** 1 Department of Urology, Lu’an People’s Hospital of Anhui Province, Lu’an Hospital of Anhui Medical University, No.21, Wanxi West Road, Lu’an, 237000 Anhui China; 2 The First Affiliated Hospital of Anhui Medical University, Anhui Public Health Clinical Center, An Hui Sheng, China; 3 Department of Orthopaedic, Lu’an People’s Hospital of Anhui Province, Lu’an Hospital of Anhui Medical University, Lu’an, 237000 China; 4 Department of Pharmacy, Lu’an People’s Hospital of Anhui Province, Lu’an Hospital of Anhui Medical University, Lu’an, 237000 China; 5 Department of Urology, Tianjin Institute of Urology, The Second Hospital of Tianjin Medical University, Tianjin, 300211 China

**Keywords:** Urine exosome, lncRNA SNHG16, Diagnosis, Biomarker, Bladder cancer

## Abstract

**Objective:**

To detect the expression level of urinary exosomal lncRNA SNHG16 in patients with bladder cancer and healthy individuals and explore its clinical application value in the diagnosis of bladder cancer.

**Methods:**

Urine samples were collected from 42 patients with bladder cancer and 42 healthy volunteers who visited Lu’an Hospital of Anhui Medical University and the Second Hospital of Tianjin Medical University from January 2020 to December 2022. The expression levels of lncRNA SNHG16 in urinary exosomes of the two groups were detected by RT‒qPCR, and their correlation with clinical pathological parameters of bladder cancer patients was analysed. An Receiver Operating Characteristic(ROC) curve was drawn to analyse the diagnostic value of urinary exosomal lncRNA SNHG16 for bladder cancer and compared with urinary cytology.

**Results:**

The expression of urinary exosomal lncRNA SNHG16 in patients with bladder cancer was significantly higher (P < 0.05), and the expression level had no correlation with the age, sex, pathological T stage, pathological grade, or tumour size of bladder cancer patients (P > 0.05). The Area Under Curve(AUC) of urinary exosomal lncRNA SNHG16 in diagnosing bladder cancer was 0.791, which was superior to that of urinary cytology (AUC = 0.597).

**Conclusion:**

Urinary exosomal lncRNA SNHG16 with high expression can serve as a potential diagnostic biological marker for bladder cancer.

Bladder cancer is a highly prevalent malignant tumour worldwide, accounting for 90–95% of urothelial carcinomas. The age-standardized incidence rate of bladder cancer is 9.0 per 100,000 in men and 2.2 per 100,000 in women worldwide [1]. Despite the continuous development of treatment methods, the five-year survival rate for bladder cancer remains less than 20% [2], especially for metastatic bladder cancer [1, 3, 4]. Early diagnosis is the key to timely and effective treatment, improving the prognosis of bladder cancer and reducing the recurrence rate and mortality. Currently, cystoscopy is the gold standard for diagnosing bladder cancer, but due to its complexity and invasiveness, it is difficult to promote it widely in population screening, resulting in a considerable number of patients being diagnosed with muscle-invasive bladder cancer at the first diagnosis, missing the optimal timing for treatment and leading to a poor prognosis. Urine cytology, as one of the most common methods for auxiliary diagnosis of bladder cancer, has a sensitivity of 25-95% for detecting urothelial carcinoma [5–10]. However, it is relatively ineffective in identifying low-grade bladder tumours. Imaging methods, such as CT and ultrasound, are easy to use to judge large-volume occupying lesions, but at this point, the patient is already in the middle and late stages, missing the optimal timing for treatment. CT and ultrasound are prone to miss small lesions, which is not conducive to the early detection and treatment of tumours [11, 12]. Therefore, it is urgent to find reliable and noninvasive biomarkers to assist in the early diagnosis of bladder cancer.

LncRNAs are a family of noncoding RNAs defined as transcripts larger than 200 nucleotides. Increasing evidence suggests that the expression of lncRNAs changes in response to tumour development, which can be used for the diagnosis, progression, and prognosis of cancer. However, naked lncRNAs are susceptible to degradation by ribonucleases in bodily fluids and require protection from extracellular vesicles. Exosomes are cell-derived microvesicles with a diameter of approximately 30–150 nm that are present in almost all body fluids. Exosomal vesicles act as “carriers” and “protective shields” that wrap around lncRNAs, preventing their degradation by ribonucleases in bodily fluid circulation [13, 14]. However, the functional contents of these vesicles are not randomly loaded but rather produced differentially according to various pathological conditions [15]. Numerous studies have shown that the expression of lncRNAs in exosomes from the blood or urine of different cancer patients is correspondingly upregulated or downregulated and may be related to tumour staging and prognosis [16–20], making them potential noninvasive biomarkers. SNHG16, as a protumor lncRNA, is upregulated in various human cancers, such as lung cancer, breast cancer, colorectal cancer, and cervical cancer [21–27]. In this study, we explored the predictive value of urinary exosome-derived lncRNA SNHG16 in the diagnosis of bladder cancer.

## Data and methods

### Collection of urine specimens

Urinary specimens were collected from 42 patients with bladder cancer and 42 healthy volunteers who underwent physical examinations at Lu’an Hospital of Anhui Medical University and the Second Hospital of Tianjin Medical University between January 2020 and December 2022, defined as the bladder cancer group and control group, respectively. Informed consent was obtained from all participants, and the study was approved by the ethics committee of the Second Hospital of Tianjin Medical University. Inclusion criteria: [1] all subjects were clinically diagnosed with bladder cancer by a physician and confirmed by surgical pathology or cystoscopy biopsy; [2] had no history of radiotherapy, chemotherapy or other anticancer treatments. The exclusion criteria were as follows: [1] patients with other organ dysfunctions, such as heart, lung, liver, or kidney dysfunction; [2] patients with heart disease, diabetes, hypertension, mental disorders, organ failure, immune diseases, and traumatic diseases; and [3] patients with other systemic tumours. Tumour grading and TNM staging were performed according to the WHO staging criteria. After collecting 100 ml of urine sample for each case, preprocessing was performed within two hours, followed by centrifugation at 3000×g for 20 min to obtain the supernatant, which was stored at -80 °C for exosome extraction. The centrifugal precipitate was sent to the pathology department for urine cytology testing.

### Cell culture

Human Bladder cancer cell T24 (ATCC) and Benign urothelial cell SV-HUC-1 (ATCC) were cultured, subcultured and cryo-preserved in Tianjin Institute of Urology. All the cells were cultured in a saturated humidity incubator at 37℃ and 5% CO_2_.

### Enrichment of urinary extracellular vesicles

The preprocessed urine samples were subjected to differential centrifugation using an ultrahigh-speed centrifuge (Beckman Coulter, USA) at 4 °C and 17,000 × g for 30 min. The supernatant was collected and subjected to a second centrifugation at 4 °C and 200,000 × g for 70 min. The supernatant was discarded, and 100 µl of PBS was added to resuspend the extracellular vesicles. The resuspended solution was aliquoted and stored at -80 °C for further analysis.

### Extraction and reverse transcription of extracellular vesicle RNA

Total RNA was extracted from extracellular vesicles using the Exosome RNA Isolation Kit (Rengen Biosciences Co., Ltd, China). The concentration and integrity of total RNA were evaluated using a NanoDrop spectrophotometer (Thermo Fisher Scientific, Waltham, MA, USA). Purified RNA was reverse transcribed into cDNA according to the manufacturer’s instructions of the US EVERBRIGHT INC kit. The 20 µl reaction mixture contained 500 ng of template RNA, 4 µl of 5×UEIris II RT MasterMix, 1 µl of dsDNase, and RNase-free ddH2O. The mixture was briefly centrifuged and then incubated at 37 °C for 2 min, followed by incubation at 55 °C for 10 min and at 85 °C for 10 s. The reaction product was immediately stored at -20 °C for subsequent experiments.

### RT-qPCR analysis

The cDNA templates were then subjected to qPCR using SYBR Green Master (Roche, Switzerland). The following primers were designed by primer 5 and synthesized by GenePharma(China):SNHG16 forward primer: 5’-AATCGCCATGCGTTCTTTGG-3’; SNHG16 reverse primer: 5’-CAATCCTTGCAGTCCCATCG-3’; 18 S forward primer: 5’-GTAACCCGTTGAACCCCATT-3’; 18 S reverse primer: 5′CCATCCAATCGGTAGTAGCG–3’; 18 S was used as a reference gene. The 2 − ΔΔCt method was used to calculate the relative expression level. Experiments were repeated 3 times.

### Urine cytology

Urine samples were collected before cystoscopic examination and any other treatments, and centrifuged at 1300 g for 10 min. The sediments were used for cytological analysis, and the diagnosis was confirmed by two cytopathologists.

### Transmission electron microscopy (TEM)

Transmission electron microscopyIsolated exosomes were first resuspended in PBS, and then a 15 µL aliquot was absorbed onto carbon-coated Cu grids for 1 min.Subsequently, the grids were dyed using 15 µ L of 2.0% uranyl acetate for 1 min and allowed to dry for 15 min. The morphologyof isolated exosomes was identified by transmission electron microscopy (TEM; G2 spititi FEI; Tecnai).

### Nanoparticle tracking analysis(NTA)

The size distribution of exosomes was determined usinga Delsa Nano Analyzer (DelsaNano, Beckman Coulter,Brea, CA, USA). The capture settings and analysis settings were performed manually according to the manufacturer’s instructions.

### Western blots

Protein were prepared with a detergent buffer, and the protein concentration was determined using the bicinchoninic acid (BCA) protein assay (Solarbio, Beijin, China). Equal amounts of protein samples were separated by a 12% gel using sodium dodecyl sulfate-polyacrylamide gel electrophoresis(SDS-PAGE) and transferred onto PVDF membranes (Millipore, Billerica, MA, USA). The membranes were probed with anti-TSG101 (GeneTex, USA), anti-CD63 (Immunoway,USA) antibodies overnight at 4˚C. Immune complexes were detected by enhanced chemiluminescence (Proteintech, Chicago,USA).

### Statistical analysis

All statistical analyses, ROC curve plotting and graphing were performed using SPSS 17.0 (IBM, SPSS, Chicago, IL, USA) and GraphPad Prism 5.0 (GraphPad Software, La Jolla, CA, USA). The Kolmogorov‒Smirnov test was used to analyse the distribution of each group of samples. Since the data did not follow a normal distribution, they are presented as the median (interquartile range). The nonparametric Mann‒Whitney U test was used to compare the expression levels of lncRNAs between the two groups. A two-sided P value of less than 0.05 was considered statistically significant.

## Results

### Identification of urinary exosomes in bladder cancer

**Fig. 1:**
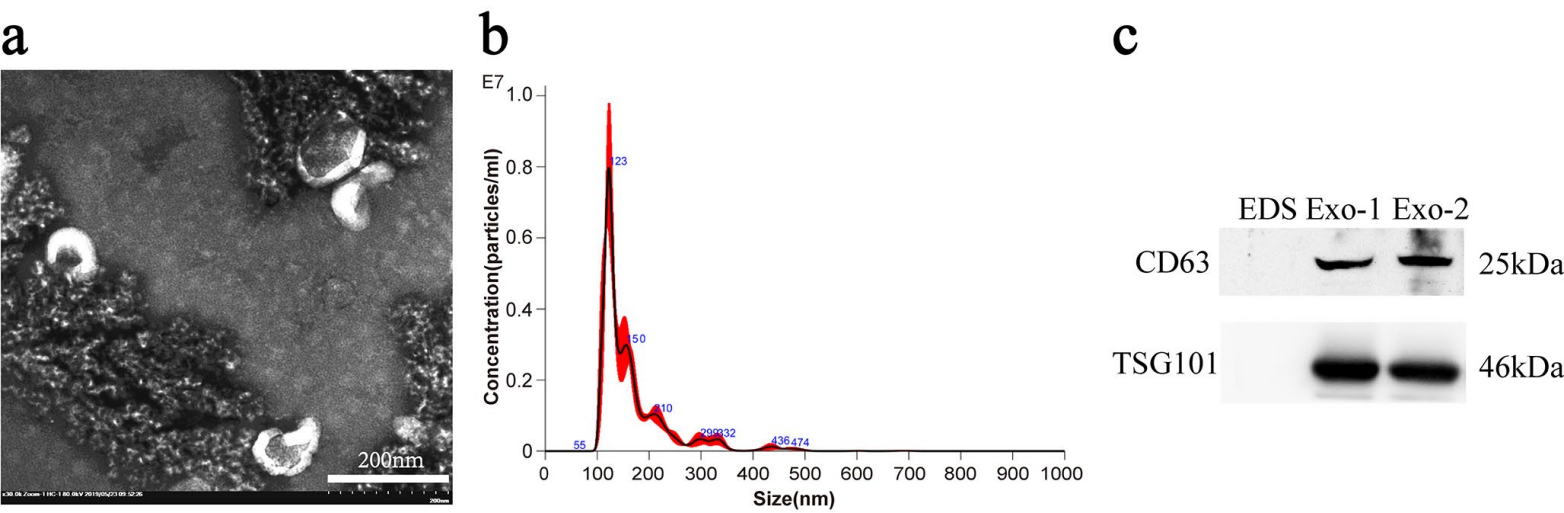
The identification of exosomes was performed as follows: TEM (**a)**, NTA (**b)**, and Western blot **(c)**. Exo-1 and Exo-2 represent urinary exosomes of bladder cancer group and control group, respectively

TEM showed exosomes have a diameter of 30–150 nm with a cup-shaped membrane (Fig. 1a**)**. NTA detected exosome particle size distribution, and the results showed that the highest particle size distribution peak in the exosome suspension was 123 nm (Fig. 1b). Western blot analysis of exosome marker proteins showed clear CD63 and TSG101 protein bands, but no protein expression was found in the exosome-depleted supernatant(EDS) (Fig. 1c). All the above results suggested that the enriched extracellular vesicles had the characteristics of exosomes.

### Differential Analysis of lncRNA SNHG16 expression in urine exosomes between the two groups

**Fig. 2:**
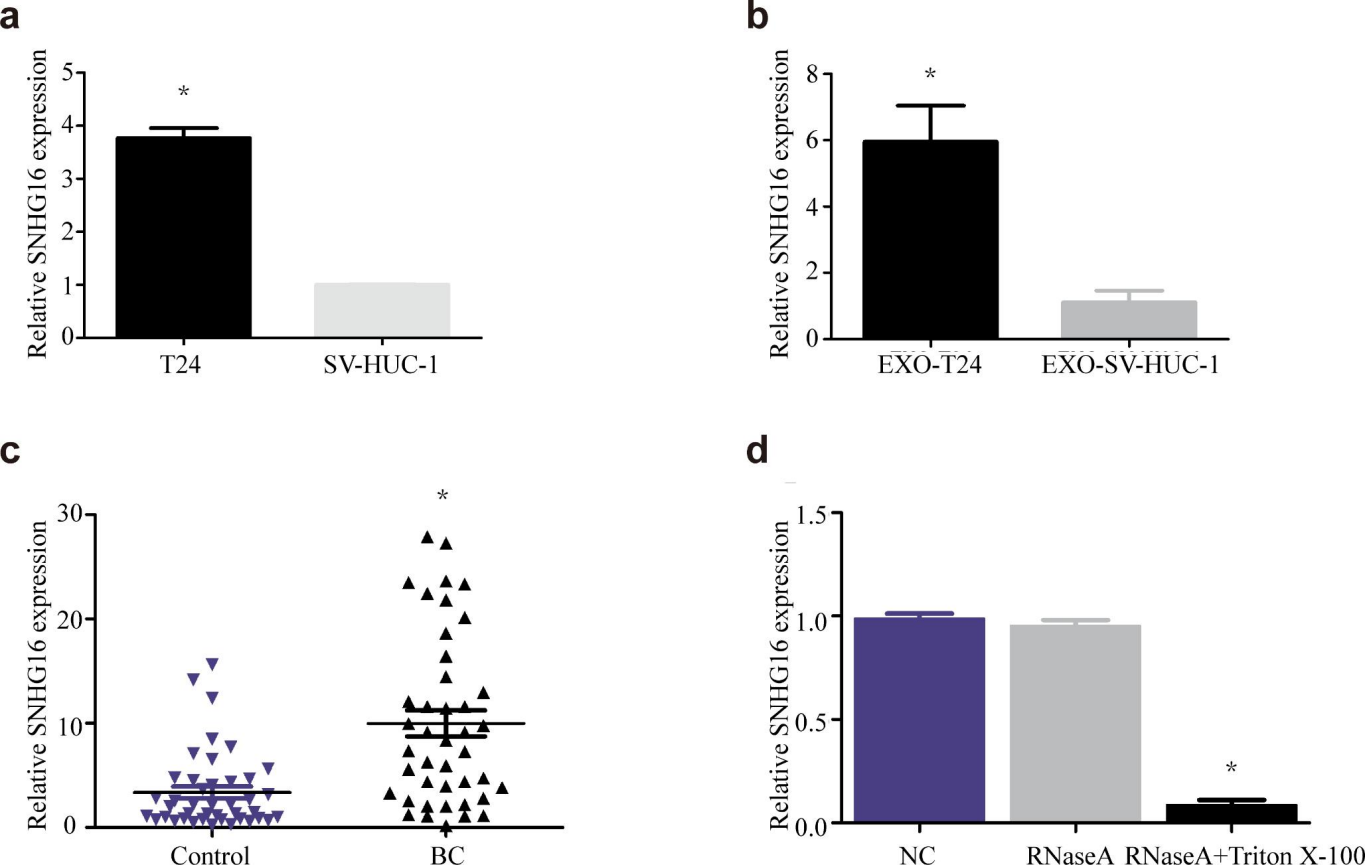
Differential expression analysis of urinary exosomal lncRNA SNHG16 in the two groups. The expression of lncRNA SNHG16 was higher in T24 cells than in SV-HUC-1 cells (**a**), higher in exosomes derived from T24 cells than in those derived from SV-HUC-1 cells (**b**), and higher in exosomes from bladder cancer patients than in the control group (**c**). RT‒qPCR showed that lncRNA SNHG16 was stably expressed in exosome vesicles (**d**). *, P value＜ 0.05

To determine whether there is a difference in the expression of lncRNA SNHG16 in urine exosomes between patients with bladder cancer and healthy controls, we first used RT‒qPCR to compare the differences in lncRNA SNHG16 expression between the bladder cancer cell line T24 and the benign urinary tract epithelial cell line SV-HUC-1, as well as between exosomes derived from these two cell sources. The results show that the expression of lncRNA SNHG16 was higher in T24 cells than in SV-HUC-1 cells (Fig. 2a) and higher in exosomes derived from T24 cells than in exosomes derived from SV-HUC-1 cells (Fig. 2b). Since bladder tumours originate from the urinary tract epithelium and the tumour is directly exposed to urine, we hypothesize that exosomes secreted by the tumour with high expression of lncRNA SNHG16 will directly enter urine. Therefore, the expression of lncRNA SNHG16 in urine exosomes of bladder cancer patients may be higher than that of healthy controls. Next, we used RT‒qPCR to detect the relative expression levels of urine exosomal lncRNA SNHG16 in the two groups. The results show that the expression levels of lncRNA SNHG16 in urine exosomes of the bladder cancer group and control group were 3.49 (2.17, 4.77) and 1.54 (0.86, 2.49), respectively, and the difference was statistically significant (P < 0.001, Fig. 2c), which means that urine exosomal lncRNA SNHG16 may become a potential diagnostic biomarker for bladder cancer. Finally, to investigate whether the expression of lncRNA SNHG16 in exosomes was stable, we performed an experiment using RT‒qPCR. The enriched urine exosome suspension was divided into three groups: the first group was the negative control group, the second group was treated with 5 µg RNaseA, and the third group was treated with 5% Triton X-100 and 5 µg RNaseA. The results show that compared with the control group, the expression of lncRNA SNHG16 in the RNaseA group did not change significantly, possibly due to the protection of exosomes. However, in the RNaseA + 5% Triton X-100 group, Triton X-100 destroyed the exosome membrane structure, leading to RNA degradation by RNaseA and a sharp decrease in lncRNA SNHG16 expression (Fig. 2d). Therefore, we believe that lncRNA SNHG16 mainly exists stably inside the exosome vesicles, with exosome vesicles as a “protective umbrella”. Therefore, urine exosomal lncRNA SNHG16 may be a stable diagnostic biomarker for bladder cancer.

### Relationship between urinary exosomal lncRNA SNHG16 levels and clinical pathological characteristics of bladder cancer patients

According to the different clinical pathological characteristics of the tumours, the bladder cancer patients were grouped by age, sex, tumour T stage, pathological grade, and maximum tumour diameter, and the expression levels of urinary exosomal lncRNA SNHG16 were compared and analysed for differences in these clinical pathological parameters. The results showed no significant differences among the groups (P > 0.05) (Table 1).

**Table 1:** Correlations between exosomal lncRNA concentrations and clinicopathological characteristics of patients with BC [median (interquartile range)]

Parameters	Total cases	Expression level	P-value
Age			
<60	11	2.81(1.14,4.58)	0.350
≥ 60	31	4.13(2.45,4.88)	
Sex			
Male	33	3.11(2.17,4.66)	0.587
Female	9	4.13(2.34,5.55)	
Tumour stage			
NMIBC(Ta-T1)	18	3.1(1.91,4.25)	0.232
MIBC(T2-T4)	24	4.33(2.48,5.63)	
Tumour grade			
Low grade	13	2.81(1.81,5.41)	0.936
High grade	29	3.84(2.48,4.66)	
Tumor size(cm)			
< 3.0	19	3.84(2.04,4.88)	0.870
≥ 3.0	23	3.14(2.50,4.73)	

### Diagnostic value of the urinary exosomal lncRNA SNHG16 expression level for bladder cancer

**Fig. 3:**
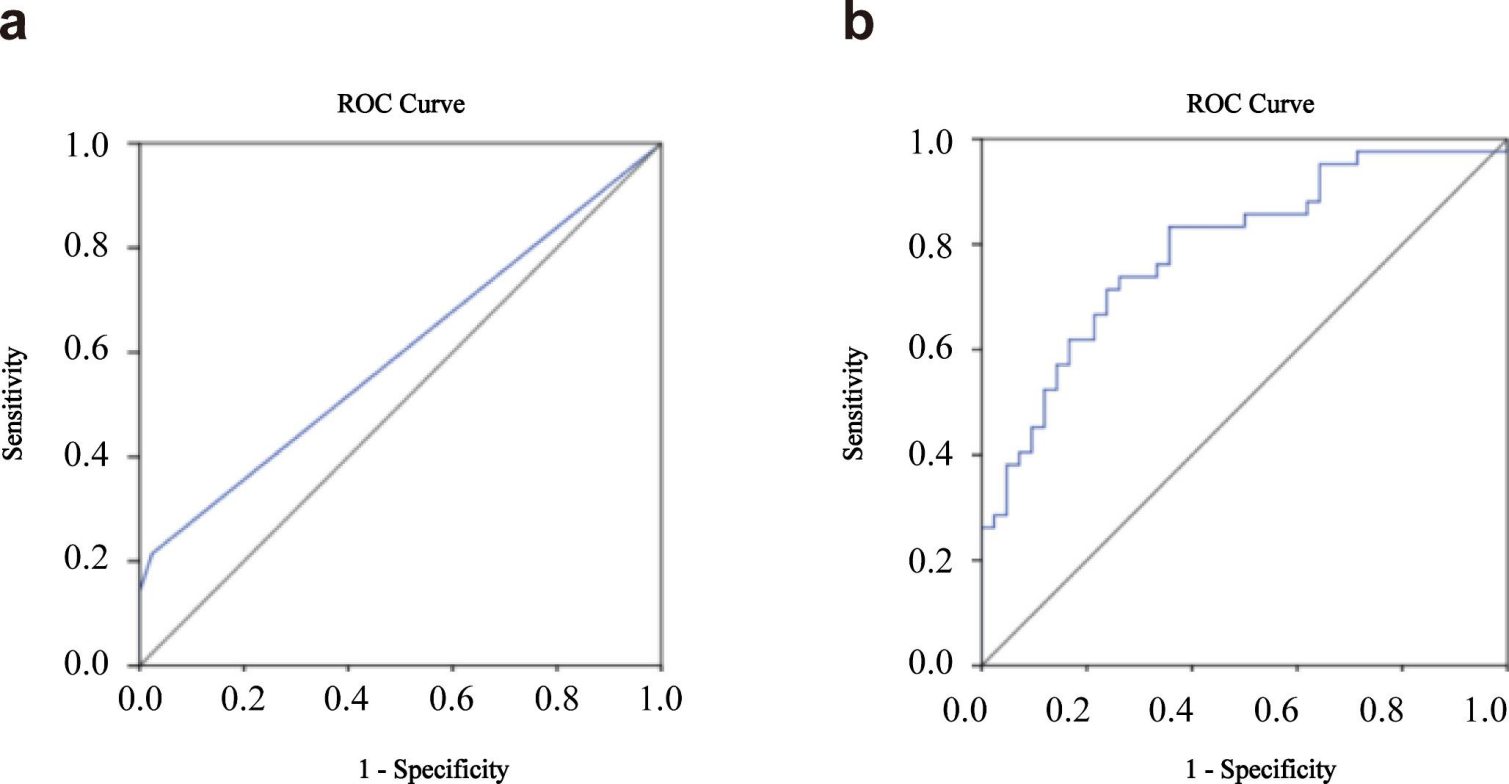
Diagnostic value of the urine extracellular vesicle lncRNA SNHG16 expression level in bladder cancer. ROC curve analysis was performed to evaluate the diagnostic performance of urine cytology in bladder cancer (**a**) and urine extracellular vesicle lncRNA SNHG16 in bladder cancer (**b**)

Currently, noninvasive detection methods used for bladder cancer diagnosis in clinical practice mostly involve urinary cytology. Therefore, we compared the results of urinary cytology in the two groups and used ROC curve analysis to evaluate the diagnostic efficacy of urinary cytology for bladder cancer, which showed an AUC of 0.597 (95% CI: 0.475-0.719, sensitivity = 21.40%, specificity = 97.6%, Fig. 3a). Further analysis showed that the AUC of urinary exosomal lncRNA SNHG16 for diagnosing bladder cancer was 0.791 (95% CI: 0.695‐0.887, sensitivity = 61.90%, specificity = 83.3%, Fig. 3b), which was significantly better than that of urinary cytology.

## Discussion

lncRNAs can serve as potential diagnostic, metastatic, and prognostic markers for bladder cancer. For example, the expression of lncRNAs such as TUC338 and PVT1 can be used as biomarkers for early diagnosis of bladder cancer [28, 29], while the expression of PCAT6, NRON, GAS6-AS2, SNHG3, lncRNA TP73-AS1, and LINC00641 can predict poor prognosis of bladder cancer [30–35]. In addition, lncRNAs can also serve as potential biomarkers for bladder cancer metastasis, such as DLX6-AS1, which is upregulated in bladder cancer tissue and cell lines and is associated with TNM stage progression, lymph node metastasis, and distant metastasis [36]. lncRNA SNHG16 is located on chromosome 17q25.1 and was first identified as an oncogene of neuroblastoma [37]. Cao [38] confirmed that the expression of lncRNA SNHG16 is significantly higher in bladder cancer tissue than in adjacent normal tissue and is associated with tumour metastasis, pathological staging, and overall survival of patients. Inhibition of SNHG16 expression can significantly suppress the proliferation, migration, and invasion of bladder cancer cells, and promote cell apoptosis, possibly through inhibition of the Wnt/β-catenin pathway [39].

LncRNA is susceptible to degradation by ribonucleases in bodily fluids and is typically found free-floating throughout the body through exosome transport. Exosome-carried lncRNAs have many advantages as tumour markers, including: (1) good specificity, sensitivity, and noninvasiveness; (2) noncoding RNAs packaged in exosomes can escape degradation by ribonucleases, making them stable; and (3) convenient sampling, with blood and urine being the most common sources of exosomes. Studies have reported that the expression of exosome-lncRNA H19 in serum of bladder cancer patients is upregulated [16], and exosome-lncRNA PTENP1 in plasma is downregulated [40], both of which can serve as good tumour markers for bladder cancer diagnosis. However, obtaining urine samples is more convenient and less painful for patients compared to blood samples. Research has shown that bladder cancer cells can secrete exosomes into urine, and stable lncRNA has been found in exosomes [41]. Zhan et al. [19] proposed a combination of lncRNAs derived from urine exosomes for bladder cancer diagnosis and recurrence prediction. The combination of three lncRNAs (MALAT1, PCAT-1, and SPRY4-IT1) had an area under the ROC curve (AUC) of 0.854 for bladder cancer diagnosis, which was significantly higher than that for urine cytology (0.619). In addition, the upregulation of PCAT-1 and MALAT1 was correlated with poor nonmuscle invasive bladder cancer-free survival. Another study showed that exosome-lncRNAs ANRIL and PCAT-1 in urine could serve as potential diagnostic biomarkers for bladder cancer, with AUCs of 0.7229 (sensitivity of 46.67% and specificity of 87.5%) and 0.7292 (sensitivity of 43.33% and specificity of 87.5%), respectively [42].

Currently, research has reported that the lncRNA SNHG16 is highly expressed in the serum exosomes of bladder cancer patients and can be used to differentiate between bladder cancer cases and healthy individuals [43]. This project aimed to investigate the feasibility of urinary exosomal SNHG16 as a diagnostic biomarker for bladder cancer. The results show that the expression of lncRNA SNHG16 was higher in T24 cells than in SV-HUC-1 cells, and its expression in exosomes derived from T24 cells was also higher than that from SV-HUC-1 cells. Since bladder cancer tumours are directly exposed to urine, it is inferred that exosomes with high expression of lncRNA SNHG16 secreted by bladder cancer may directly enter urine. Therefore, the expression of lncRNA SNHG16 in urinary exosomes of bladder cancer patients may be higher than that of healthy individuals. Further research showed that there was a statistically significant difference in the expression level of urinary exosomal lncRNA SNHG16 between the bladder cancer group and the control group (P < 0.001). This suggests that urinary exosomal lncRNA SNHG16 may be a noninvasive and stable diagnostic biomarker for bladder cancer. Finally, we used the relative expression value of RT‒qPCR to plot the ROC curve, and the results show that the diagnostic performance of urinary exosomal lncRNA SNHG16 for bladder cancer (AUC = 0.791, 95% CI: 0.695–0.887, sensitivity = 61.90%, specificity = 83.3%) was better than that of urinary cytology (AUC = 0.597, 95% CI: 0.475–0.719, sensitivity = 21.40%, specificity = 97.6%). In the data analysis, we did not find any correlation between the expression level of urinary exosomal lncRNA SNHG16 and age, sex, pathological T stage, pathological grade, or tumour size of bladder cancer patients (P > 0.05), which may require analysis of more samples to uncover hidden associations.

## Conclusion

In summary, we believe that urinary exosomal lncRNA SNHG16 can serve as a noninvasive and reliable diagnostic biomarker for bladder cancer, but further large-scale and multicentre studies are still needed to discover whether the expression of urinary exosomal lncRNA SNHG16 is associated with the survival prognosis, pathological staging, and distant metastasis of bladder cancer patients.

## Data Availability

The datasets generated during and/or analysed during the current study are available from the corresponding author on reasonable request.

## Abbreviations

AUC: Transmission electron microscopy
TEM: Transmission electron microscopy
NTA: Nanoparticle tracking analysis
ROC: Receiver Operating Characteristic
EDS: Exosome-depleted supernatant

## References

[1] Babjuk M, Bohle A, Burger M, Capoun O, Cohen D, Comperat EM (2017). EAU Guidelines on Non-Muscle-invasive Urothelial Carcinoma of the bladder: Update 2016. Eur Urol.

[2] Aggen DH, Drake CG (2017). Biomarkers for immunotherapy in bladder cancer: a moving target. J Immunother Cancer.

[3] Kubota Y, Nakaigawa N (2016). Committee for Establishment of the Clinical Practice Guideline for the management of bladder C, the japanese Urological A. essential content of evidence-based clinical practice guidelines for bladder cancer: the japanese Urological Association 2015 update. Int J Urol.

[4] Clark PE, Spiess PE, Agarwal N, Bangs R, Boorjian SA, Buyyounouski MK (2016). NCCN Guidelines Insights: bladder Cancer, Version 2.2016. J Natl Compr Canc Netw.

[5] Giannopoulos A, Manousakas T, Mitropoulos D, Botsoli-Stergiou E, Constantinides C, Giannopoulou M (2000). Comparative evaluation of the BTAstat test, NMP22, and voided urine cytology in the detection of primary and recurrent bladder tumors. Urology.

[6] Gregoire M, Fradet Y, Meyer F, Tetu B, Bois R, Bedard G (1997). Diagnostic accuracy of urinary cytology, and deoxyribonucleic acid flow cytometry and cytology on bladder washings during followup for bladder tumors. J Urol.

[7] Grossman HB (1998). New methods for detection of bladder cancer. Semin Urol Oncol.

[8] Pode D, Shapiro A, Wald M, Nativ O, Laufer M, Kaver I (1999). Noninvasive detection of bladder cancer with the BTA stat test. J Urol.

[9] Ramakumar S, Bhuiyan J, Besse JA, Roberts SG, Wollan PC, Blute ML (1999). Comparison of screening methods in the detection of bladder cancer. J Urol.

[10] Zippe C, Pandrangi L, Potts JM, Kursh E, Novick A, Agarwal A (1999). NMP22: a sensitive, cost-effective test in patients at risk for bladder cancer. Anticancer Res.

[11] Crozier J, Papa N, Perera M, Ngo B, Bolton D, Sengupta S (2019). Comparative sensitivity and specificity of imaging modalities in staging bladder cancer prior to radical cystectomy: a systematic review and meta-analysis. World J Urol.

[12] Schulz GB, Gresser EK, Casuscelli J, Strittmatter F, Tritschler S, Karl A (2019). [Value of imaging in upper urinary tract tumors]. Urologe A.

[13] Yang H, Fu H, Xu W, Zhang X (2016). Exosomal non-coding RNAs: a promising cancer biomarker. Clin Chem Lab Med.

[14] Li Q, Shao Y, Zhang X, Zheng T, Miao M, Qin L (2015). Plasma long noncoding RNA protected by exosomes as a potential stable biomarker for gastric cancer. Tumour Biol.

[15] Santangelo L, Giurato G, Cicchini C, Montaldo C, Mancone C, Tarallo R (2016). The RNA-Binding protein SYNCRIP is a component of the hepatocyte Exosomal Machinery Controlling MicroRNA sorting. Cell Rep.

[16] Wang J, Yang K, Yuan W, Gao Z (2018). Determination of serum exosomal H19 as a noninvasive biomarker for bladder Cancer diagnosis and prognosis. Med Sci Monit.

[17] Tao Y, Tang Y, Yang Z, Wu F, Wang L, Yang L (2020). Exploration of serum exosomal LncRNA TBILA and AGAP2-AS1 as promising biomarkers for diagnosis of Non-Small Cell Lung Cancer. Int J Biol Sci.

[18] Cai C, Zhang H, Zhu Y, Zheng P, Xu Y, Sun J (2019). Serum Exosomal Long Noncoding RNA pcsk2-2:1 as a potential Novel Diagnostic Biomarker for gastric Cancer. Onco Targets Ther.

[19] Zhan Y, Du L, Wang L, Jiang X, Zhang S, Li J (2018). Expression signatures of exosomal long non-coding RNAs in urine serve as novel non-invasive biomarkers for diagnosis and recurrence prediction of bladder cancer. Mol Cancer.

[20] Ding XZ, Zhang SQ, Deng XL, Qiang JH (2021). Serum exosomal lncRNA DLX6-AS1 is a Promising Biomarker for Prognosis Prediction of Cervical Cancer. Technol Cancer Res Treat.

[21] Chen L, Qiu CH, Chen Y, Wang Y, Zhao JJ, Zhang M (2020). LncRNA SNHG16 drives proliferation, migration, and invasion of lung cancer cell through modulation of miR-520/VEGF axis. Eur Rev Med Pharmacol Sci.

[22] Du SM (2020). The SNHG16/miR-30a axis promotes breast cancer cell proliferation and invasion by regulating RRM2. Neoplasma.

[23] Ke D, Wang Q, Ke S, Zou L, Wang Q, Long-Non Coding (2020). RNA SNHG16 supports Colon cancer cell growth by modulating miR-302a-3p/AKT Axis. Pathol Oncol Res.

[24] Tao L, Wang X, Zhou Q (2020). Long noncoding RNA SNHG16 promotes the tumorigenicity of cervical cancer cells by recruiting transcriptional factor SPI1 to upregulate PARP9. Cell Biol Int.

[25] Xia W, Liu Y, Cheng T, Xu T, Dong M, Hu X (2021). Extracellular vesicles carry lncRNA SNHG16 to promote metastasis of breast Cancer cells via the miR-892b/PPAPDC1A Axis. Front Cell Dev Biol.

[26] Yu L, Chen D, Song J (2020). LncRNA SNHG16 promotes non-small cell lung cancer development through regulating EphA2 expression by sponging miR-520a-3p. Thorac Cancer.

[27] Xiang Z, Huang G, Wu H, He Q, Yang C, Dou R (2022). SNHG16 upregulation-induced positive feedback loop with YAP1/TEAD1 complex in Colorectal Cancer cell lines facilitates liver metastasis of colorectal cancer by modulating CTCs epithelial-mesenchymal transition. Int J Biol Sci.

[28] Li G, Zhang Y, Mao J, Hu P, Chen Q, Ding W (2019). lncRNA TUC338 is a potential diagnostic biomarker for bladder cancer. J Cell Biochem.

[29] Yu C, Longfei L, Long W, Feng Z, Chen J, Chao L (2019). LncRNA PVT1 regulates VEGFC through inhibiting miR-128 in bladder cancer cells. J Cell Physiol.

[30] Zhang D, Du D, Yi S, Li X (2020). LncRNA PCAT6: a potential biomarker for diagnosis and prognosis of bladder cancer. Ann Diagn Pathol.

[31] Xiong T, Huang C, Li J, Yu S, Chen F, Zhang Z (2020). LncRNA NRON promotes the proliferation, metastasis and EMT process in bladder cancer. J Cancer.

[32] Rui X, Wang L, Pan H, Gu T, Shao S, Leng J (2019). LncRNA GAS6-AS2 promotes bladder cancer proliferation and metastasis via GAS6-AS2/miR-298/CDK9 axis. J Cell Mol Med.

[33] Dai G, Huang C, Yang J, Jin L, Fu K, Yuan F (2020). LncRNA SNHG3 promotes bladder cancer proliferation and metastasis through miR-515-5p/GINS2 axis. J Cell Mol Med.

[34] Tuo Z, Zhang J, Xue W (2018). LncRNA TP73-AS1 predicts the prognosis of bladder cancer patients and functions as a suppressor for bladder cancer by EMT pathway. Biochem Biophys Res Commun.

[35] Li Z, Hong S, Liu Z (2018). LncRNA LINC00641 predicts prognosis and inhibits bladder cancer progression through miR-197-3p/KLF10/PTEN/PI3K/AKT cascade. Biochem Biophys Res Commun.

[36] Guo J, Chen Z, Jiang H, Yu Z, Peng J, Xie J (2019). The lncRNA DLX6-AS1 promoted cell proliferation, invasion, migration and epithelial-to-mesenchymal transition in bladder cancer via modulating Wnt/beta-catenin signaling pathway. Cancer Cell Int.

[37] Yu M, Ohira M, Li Y, Niizuma H, Oo ML, Zhu Y (2009). High expression of ncRAN, a novel non-coding RNA mapped to chromosome 17q25.1, is associated with poor prognosis in neuroblastoma. Int J Oncol.

[38] Cao X, Xu J, Yue D (2018). LncRNA-SNHG16 predicts poor prognosis and promotes tumor proliferation through epigenetically silencing p21 in bladder cancer. Cancer Gene Ther.

[39] Feng F, Chen A, Huang J, Xia Q, Chen Y, Jin X (2018). Long noncoding RNA SNHG16 contributes to the development of bladder cancer via regulating miR-98/STAT3/Wnt/beta-catenin pathway axis. J Cell Biochem.

[40] Zheng R, Du M, Wang X, Xu W, Liang J, Wang W (2018). Exosome-transmitted long non-coding RNA PTENP1 suppresses bladder cancer progression. Mol Cancer.

[41] Beckham CJ, Olsen J, Yin PN, Wu CH, Ting HJ, Hagen FK (2014). Bladder cancer exosomes contain EDIL-3/Del1 and facilitate cancer progression. J Urol.

[42] Abbastabar M, Sarfi M, Golestani A, Karimi A, Pourmand G, Khalili E (2020). Tumor-derived urinary exosomal long non-coding RNAs as diagnostic biomarkers for bladder cancer. EXCLI J.

[43] Zhang S, Du L, Wang L, Jiang X, Zhan Y, Li J (2019). Evaluation of serum exosomal LncRNA-based biomarker panel for diagnosis and recurrence prediction of bladder cancer. J Cell Mol Med.

